# A Cost-Effective and Sensitive Method for the Determination of Lincomycin in Foods of Animal Origin Using High-Performance Liquid Chromatography

**DOI:** 10.3390/molecules29133054

**Published:** 2024-06-27

**Authors:** Minqi Ye, Limin Hou, Zongpei Jiang, Xueyan Sun, Liangzhu Chen, Binghu Fang

**Affiliations:** 1 College of Veterinary Medicine, South China Agricultural University, Guangzhou 510642, Chinahlm@scau.edu.cn (L.H.); jzp@scau.edu.cn (Z.J.);; 2 Guangdong Wenshi Dahuanong Biotechnology Co., Ltd., Yunfu 527399, China

**Keywords:** LIN residues, high-performance liquid chromatography, edible tissues, derivatization

## Abstract

Background: Lincomycin (LIN) is extensively used for treating diseases in livestock and promoting growth in food animal farming, and it is frequently found in both the environment and in food products. Currently, most of the methods for detecting lincomycin either lack sensitivity and precision or require the use of costly equipment such as mass spectrometers. Result: In this study, we developed a reliable high-performance liquid chromatography-ultraviolet detection (HPLC-UVD) method and used it to detect LIN residue in 11 types of matrices (pig liver and muscle; chicken kidney and liver; cow fat, liver and milk; goat muscle, liver and milk; and eggs) for the first time. The tissue homogenates and liquid samples were extracted via liquid–liquid extraction, and subsequently purified and enriched via sorbent and solid phase extraction (SPE). After nitrogen drying, the products were derivatized with *p*-toluene sulfonyl isocyanic acid (PTSI) (100 µL) for 30 min at room temperature. Finally, the derivatized products were analyzed by HPLC at 227 nm. Under the optimized conditions, the method displayed impressive performance and demonstrated its reliability and practicability, with a limit of detection (LOD) and quantification (LOQ) of LIN in each matrix of 25–40 μg/kg and 40–60 μg/kg, respectively. The recovery ranged from 71.11% to 98.30%. Conclusions: The results showed that this method had great selectivity, high sensitivity, satisfactory recovery and cost-effectiveness—fulfilling the criteria in drug residue and actual detection requirements—and proved to have broad applicability in the field of detecting LIN in animal-derived foods.

## 1. Introduction

Lincomycin (LIN), a biosynthetic antibiotic among lincosamides, inhibits protein synthesis by binding to 50S subunit ribosomes and disrupting the peptidyl transferase reaction [1]. It primarily targets various anaerobic and gram-positive bacteria. LIN is a common medication for various animal diseases in animal production, such as piglets’ diarrhea after weaning, chickens’ intestinal inflammation, and cows’ breast infection [2,3]. In addition, LIN is also used as a growth promoter in some countries. As reported, 125 tons of lincosamides were sold in the USA in 2018, accounting for approximately 2% of the total antimicrobial agents used in food-producing animals [4]. In Japan, LIN sales reached 29 tons, ranking among the top ten most sold veterinary pharmaceuticals [5]. LIN is not completely broken down by animals, and it is usually released as the original compound at a rate of 32% [3,6,7]. Consequently, LIN is frequently found in the environment. Moreover, it can lead to the presence of drug residues in animal-derived foods, posing direct risks to consumers. The consumption of food with drug residues can cause adverse reactions such as gastrointestinal discomfort, nerve damage, and cardiovascular disease; and may potentially alter the intestinal community [8,9,10]. Therefore, many countries have established maximum residue limits (MRLs) for LIN in food of animal origin. For instance, the MRL for LIN in muscle tissues of all food-producing animals is set at 100 μg/kg within the European Union [11]. Thus, the development of a reliable, sensitive and cost-effective method for detecting LIN residues plays a pivotal role in regulating antibiotic abuse and ensuring food safety.

To date, many methods have been used to identify LIN residues in animal-derived food and animal feed; including fluorescent latex immunoassay (FLI), enzyme-linked immunosorbent assay (ELISA) [11,12,13], high-performance liquid chromatography (HPLC) with evaporative light-scattering detection (ELSD) [14,15,16,17,18], high-performance liquid chromatography–tandem mass spectrometry (HPLC–MS/MS) [19,20,21,22] and gas chromatography (GC) [23,24]. Compared with other methods, liquid chromatography is relatively affordable and has high sensitivity, accuracy and precision. Thus, HPLC analysis has always been the most popular and cost-effective method for accessing drug residues [25].

Like most methods, HPLC also poses a great challenge for sample pretreatment. Based on previous studies, various sample pretreatment methods have been utilized to eliminate matrix interferences in animal-derived foods. The pretreatment procedures commonly involve several steps, including extraction, defatting [26,27,28,29,30], deproteinization [31,32], various solid-phase extractions (SPE) [27,28,29,33], immunoaffinity chromatography (IAC) [32], derivatization [34], etc. Significant optimization and pretreatment steps were performed in this study, and these steps played an essential role in improving the sensitivity of LIN residue detection.

In a previous study from our laboratory, several tissues (pig fat and kidney, chicken fat and muscle, cow muscle and kidney, and goat fat and kidney) were evaluated. Therefore, the aim of this study is to optimize and establish a sensitive and cost-effective HPLC-UVD method for analyzing LIN residues in nine other edible tissues, milk and eggs; and investigated the potential of this method for LIN residue analysis. In this study, we utilized precolumn derivatization, refined pretreatment procedures and chromatographic conditions to expand the types of samples tested, ensuring successful application of the method across a wider range of animal-derived foods in different forms.

## 2. Results and Discussion

### 2.1. Optimization of the Sample Pretreatment

Pretreatment is a necessary step for improving the sensitivity of a method for drug residue analysis. Due to the complex composition of animal matrices, choosing an extractant that can simultaneously extract drugs and remove most proteins and fat is critical. In this study, the extraction procedure was optimized by altering the type and usage amount of extraction reagents, ensuring satisfactory recovery of LIN in each type of matrix.

**Figure 1:**
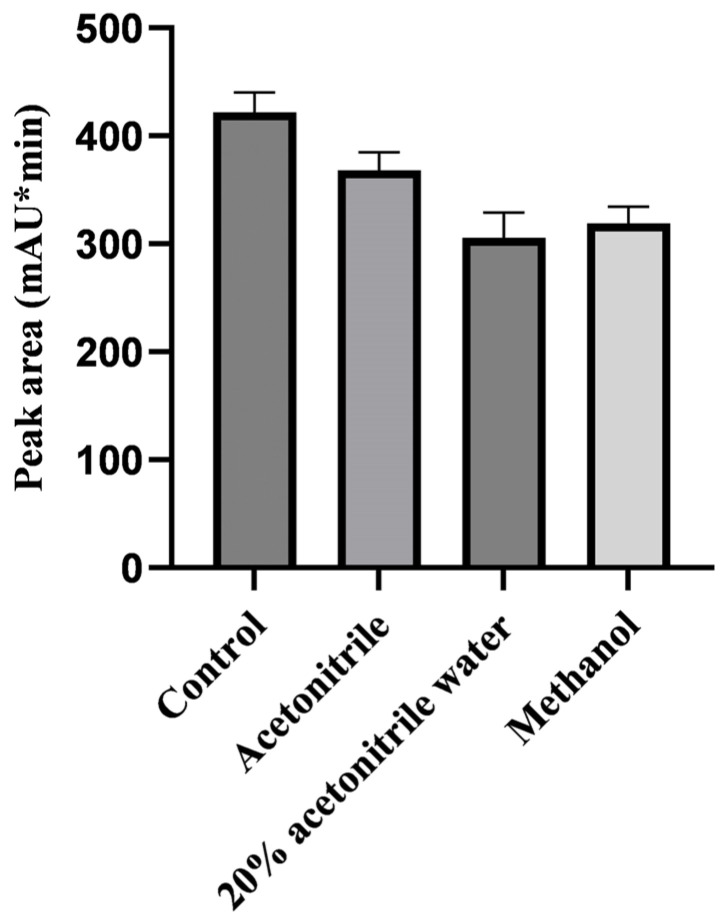
The response value of LIN derivative with spiked samples extracted by different extractants.

Both acetonitrile and methanol were the most common extraction solvents used for sample preparation. They can extract drugs with a wide range of polarities [35]. LIN is polar and soluble in water and methanol. However, methanol can introduce additional interfering compounds and proteins into the extract, increasing the difficulty of subsequent purification when used to extract LIN from samples in this study. However, acetonitrile demonstrated good solubility, strong penetration and better deproteinization. The proteins were precipitated without leaving out too much LIN. We compared the ability of three solvents (20% acetonitrile-water, acetonitrile, and methanol) to extract LIN from pig liver at 500 µg/kg and used LIN working standard solution without mixing with 5 g blank matrix sample in 50 mL centrifuge tubes as control group. The extraction recovery rate of acetonitrile was highest—more than 85%—while the recovery of the other two extractants reached approximately 75% (Figure 1). Therefore, acetonitrile extraction was ultimately selected due to its ability to extract target components effectively from animal matrices, providing a symmetrical peak shape, stable baseline, and reasonable separation time. Three samples for each treatment were analyzed. Each sample was analyzed three times by HPLC. Nine measurements were calculated for one group. Other optimization experiments were conducted in the same way.

Fat is a type of difficult matrix whose chemical properties and solubility are influenced by the type, length, degree of unsaturation, and configuration of fatty acids; which produce broad peaks that might overlap the peaks of target compound and interfere with the HPLC analysis [35]. Therefore, different sources and types of fat samples require appropriate extraction solvents. Moreover, the amount, temperature, time and frequency of extraction need to be optimized to obtain high sensitivity and recovery. Compared with pig fat and chicken fat, cow fat is harder and more easily solidified into clumps, which prevents it from being fully mixed with extraction reagents, and leading to a low recovery. Heating fat samples in a water bath before extraction was also attempted. When heated at 60 °C for 5 min, the cow fat could be completely melted. However, it was difficult for the mixture to thaw during later operations or centrifuge, which was also not convenient for analysis under cold conditions. Thus, the method of dissolving fat with reagents for softening and removing fat and fatty acid was selected, and n-hexane was chosen as the dissolving agent because it is relatively less toxic and nonpolar. Acetonitrile-water was used as an extractant for deproteination and extraction. As a result, compared with the control group—LIN working standard solution of the same concentration without cow fat in 50 mL centrifuge tubes—the recovery of cow fat showed a significant improvement from less than 40% without dissolving to more than 90%. In addition, the defatting step can sometimes be spared with this method because of the defatting of n-hexane.

**Figure 2:**
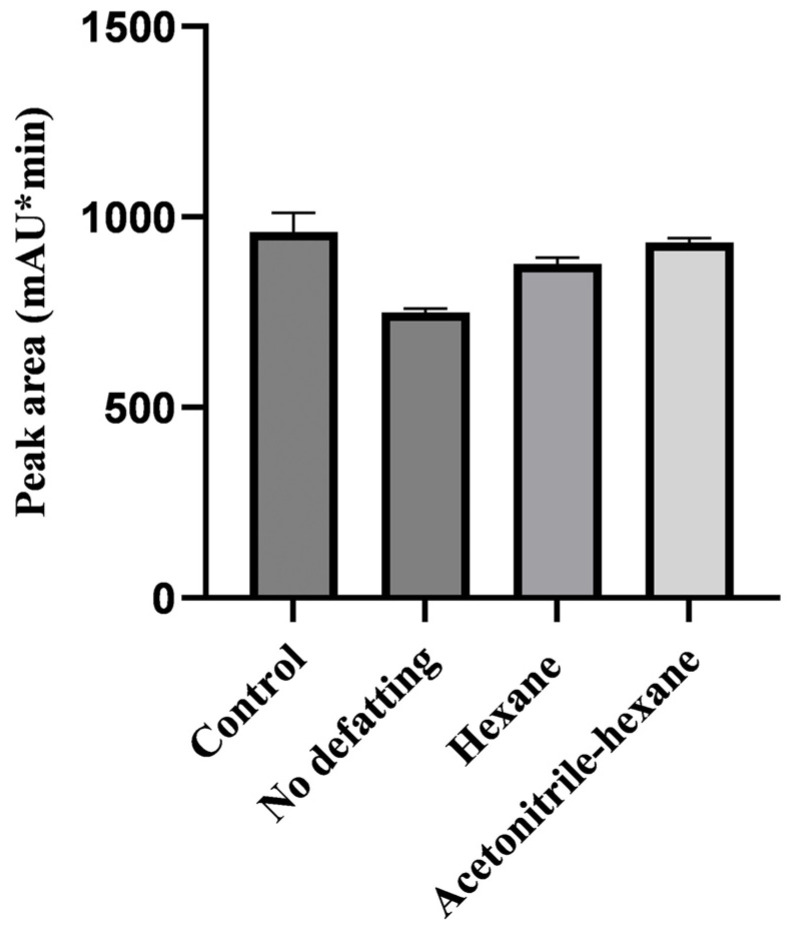
The response value of LIN derivative with spiked samples defatted by different procedures.

Pretreatment of matrices of animal origin involved a defatting step, as the presence of fats and lipids in the extract could compromise the separation and selectivity of the HPLC-UV detector for the target products. This study investigated how different defatting procedures affect the LIN recovery rate. Liver samples, one of the most complex matrices, were subjected to three different defatting procedures: no defatting, hexane defatting, and saturated acetonitrile-hexane defatting (Figure 2). LIN working standard solution without mixing with liver matrix in 50 mL centrifuge tubes was as control, for which there was no defatting steps. The results showed that the nondefatted samples had a recovery rate of 74% to 75%, and they also had more impurities and needed a longer time for purification; the hexane-defatted samples had a recovery rate of 87% to 89%, which might be due to the solubility of hexane in acetonitrile; the saturated acetonitrile-hexane defatted samples had a recovery rate of 93% to 94%, and they needed less time for purification with better impurity removal. Thus, saturated acetonitrile-hexane was chosen as the defatting agent.

**Figure 3:**
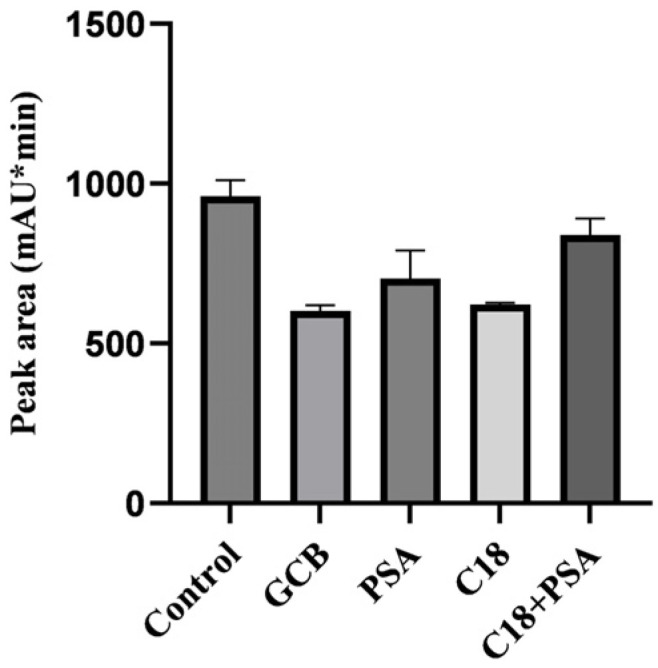
The response value of LIN derivative with spiked samples purified by different sorbants.

For sample purification and clean-up, the application of sorbents and SPE determines the effectiveness of purification and the subsequent residue analysis [36]. The sorbents used in the defatting step and the filler in SPE should selectively retain the target drug and remove interfering impurities from various matrices. In this study, activated carbon, graphitized carbon black, C18 and PSA were investigated as sorbents. Activated carbon not only eliminated impurities and pigments but also absorbed LIN, leading to low recovery. Graphitized carbon black introduced more impurities with less than 50% LIN recovered. C18 and PSA powder were commonly used to purify the sample extracts by absorbing different compounds from the sample matrix. C18 absorbs nonpolar compounds, such as lipids, while PSA removes sugars, pigments, and fatty acids by forming hydrogen bonds and weak ion exchange with polar matrix components [37]. By adjusting the amounts and ratios of these two materials, the recovery of LIN successfully increased to more than 80% in liver, kidney and egg samples; which are the most difficult samples because they are rich in pigments, lipids, metabolites and other complex impurities (Figure 3). Therefore, C18 and PSA powder were chosen as the optimal sorbents for this analysis.

### 2.2. The Selection of Derivatization Reagent and the Stability of LIN Derivatives

As LIN exhibits weak UV absorption, it needs to be derivatized and loaded with UV-absorbing groups for the HPLC-UVD analysis, which is commonly used to analyze substances with strong UV absorption. When LIN were converted into derivatives products with UV-absorbing groups, LIN would be detected and the detection sensitivity of HPLC can be dramatically improved.

**Figure 4:**
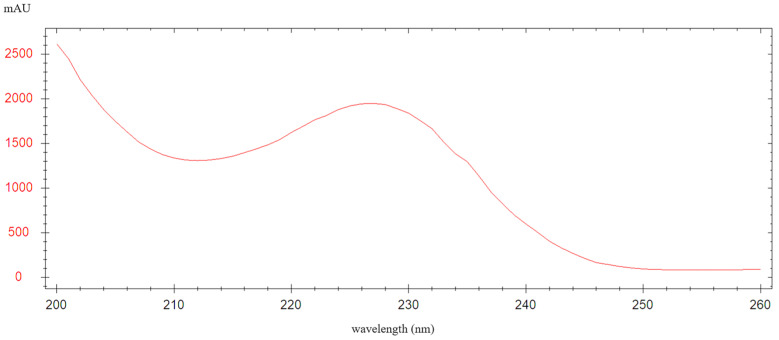
Ultraviolet scanning spectrum of LIN derivative.

PTSI is an isocyanate compound, which can react quickly with hydroxyl group and load LIN with ultraviolet absorption groups under mild conditions. Thus, PTSI was chosen for the analysis of LIN by ultraviolet (UV) detector. Meanwhile, in order to stop derivatization reaction by providing excess hydroxyl group, methanol was selected as stop solution. The reaction between PTSI and methanol was much milder than that with water. Besides, to evaluate the stability of LIN derivatives under various conditions and for various durations, we diluted standard LIN solution to concentrations of 5, 10 and 50 μg/mL using chromatographic-grade methanol. Subsequently, 100 μL of each concentration was dried under nitrogen at 45 °C and derivatized according to Section 2.4 All the samples were divided into two groups: one stored in a 4 °C refrigerator and the other stored at room temperature (25 °C). The samples were analyzed at varying time points of 0, 2, 4, 8, 12, 24, 36, 48 and 72 h. The peak areas of the derivatized products at different time points were compared to the peak area of the initial sample (0 h), providing us with an indication of the stability of the LIN derivatives under these specific conditions. The results showed that the derivatives maintained considerable stability for up to 72 h when stored at 4 °C. After exposure to 25 °C, only slight degradation occurred in 24 h, suggesting that the LIN derivatives were stable for up to 24 h at 25 °C, thus guaranteeing reliable detection (Figure 4).

### 2.3. Optimization of HPLC Conditions

Chromatographic conditions—including the type and composition of the stationary and mobile phases; the temperature, the injection volume, the flow rate and the column length and diameter—determine the efficiency, resolution, and selectivity of the separation, as well as the retention time and peak shape of the analytes. Therefore, it is necessary for this method to achieve better separation of the desired compounds from the complex mixture by optimizing these chromatographic conditions.

**Figure 5:**
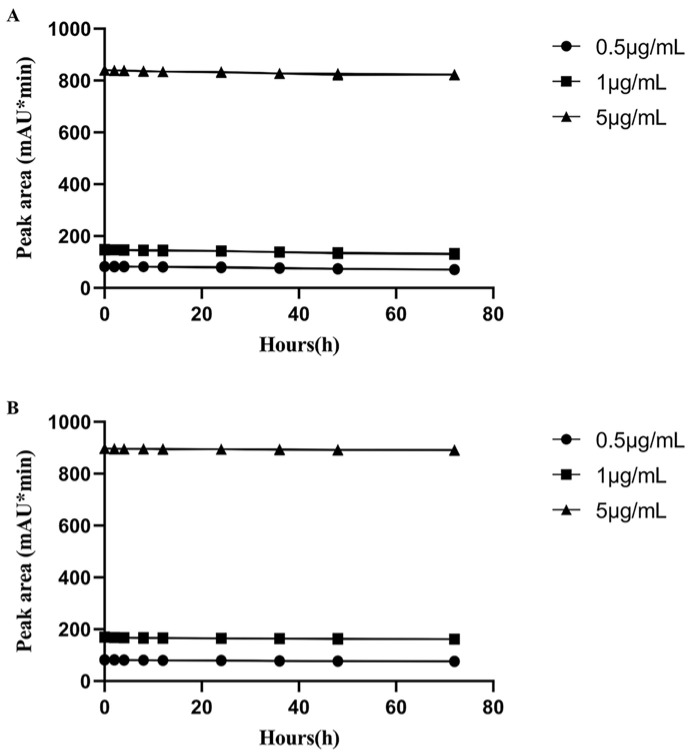
The stability of LIN derivative at 25 °C (**A**) and at 4 °C (**B**), respectively.

The detection of LIN depended on the optimal choice of the maximum UV wavelength for the derivatized product. A UV detector (Agilent, Agilent Technologies Inc., Santa Clara, CA, USA) was used to monitor the UV spectra of the lincomycin derivatives in real time and analyzed them based on their retention times. The derivatives were purified and diluted to 0.5 mg/mL. When the peak of the derivative of LIN reached 1/3 of its height, the maximum UV wavelength was measured. The results indicated that the derivative had the highest UV absorbance at 227 nm (Figure 5).

**Figure 6:**
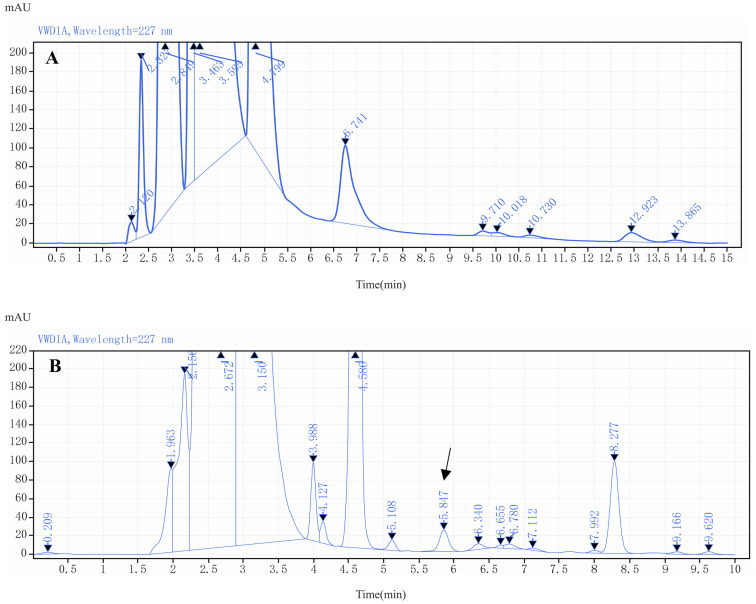
The chromatogram of LIN derivative using methanol as the organic phase in mobile phase (**A**); the chromatogram of LIN derivative using acetonitrile as the organic phase in mobile phase (**B**).

Methanol and acetonitrile are commonly used as organic solvents in HPLC [36]. The separation performance of these two solvents for LIN derivatives was examined in this study. Compared with acetonitrile, methanol resulted in inferior separation, fewer sharp peaks, and even no peaks at low analyte concentrations. However, acetonitrile gave sharper peaks and lower column pressure due to its stronger elution power, which also reduced column damage. Hence, acetonitrile was chosen as the optimal organic solvent (Figure 6).

**Figure 7:**
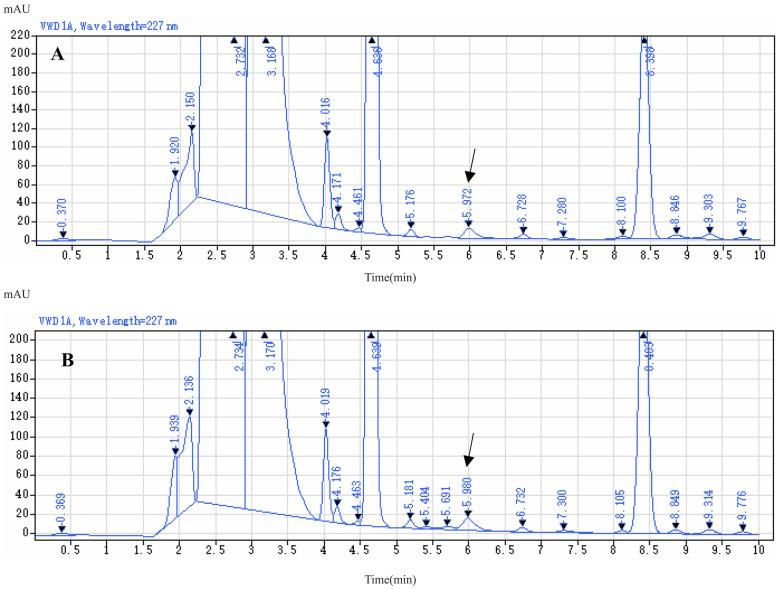
The chromatogram of LIN derivative with phosphate-phosphoric acid buffer as the aqueous phase in mobile phase (**A**); the chromatogram of LIN derivative with ammonium acetate-formic acid buffer as the aqueous phase in mobile phase (**B**).

For aqueous solvents, pure water resulted in broader peaks and lower responses for LIN derivatives. Using either 4.5 M ammonium acetate solution (0.1% formic acid) or 0.02 M potassium dihydrogen phosphate solution (0.018% phosphoric acid) as the aqueous solvent improved the responses and obtained drug peaks; but potassium dihydrogen phosphate-phosphoric acid buffer had a more stable baseline and better performance at low analyte concentrations, as shown in Figure 7. In Figure 7A, EP resolution of LIN peak was 3.39 with a peak symmetry of 0.89, and a tailing factor of 0.96, showing a good performance; which was far greater than the peak obtained with ammonium acetate-formic acid buffer as the aqueous phase in mobile phase, with a resolution of 0.79, smaller than 1.5 (Figure 7B). Thus, phosphate-phosphoric acid buffer was selected as the aqueous solvent. The proportion of the organic phase to the aqueous phase was adjusted to 60:40 so that the peak elution time was approximately 6 min.

**Figure 8:**
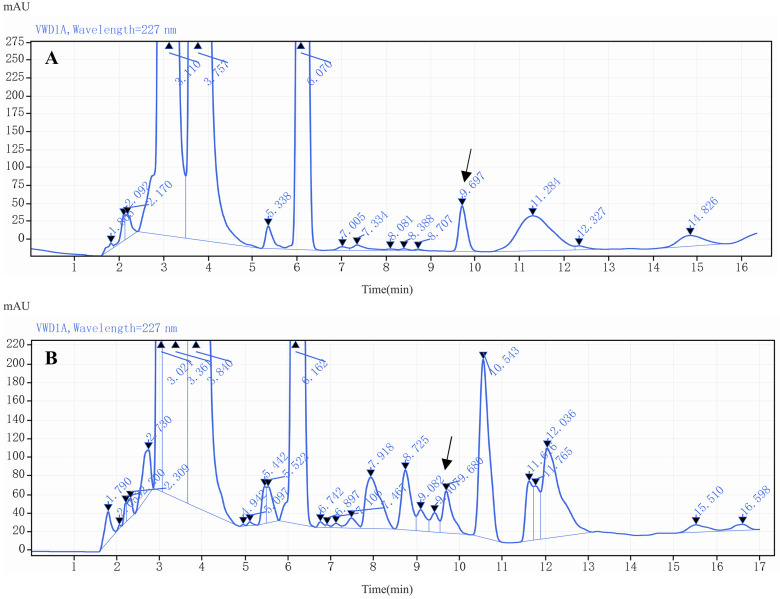
The chromatogram of LIN derivative by gradient elution (**A**); the chromatogram of spiked sample of pig liver by gradient elution (**B**).

The effect of gradient elution was tested, and the ratio and duration of the organic phase in the mobile phase varied; however, gradient elution failed to achieve better peak stability and separation efficiency in analyzing spiked samples of pig liver, with the resolution of 0.68 (Figure 8B); while prolonging the analysis time. Therefore, this approach was abandoned (Figure 8).

**Figure 9:**
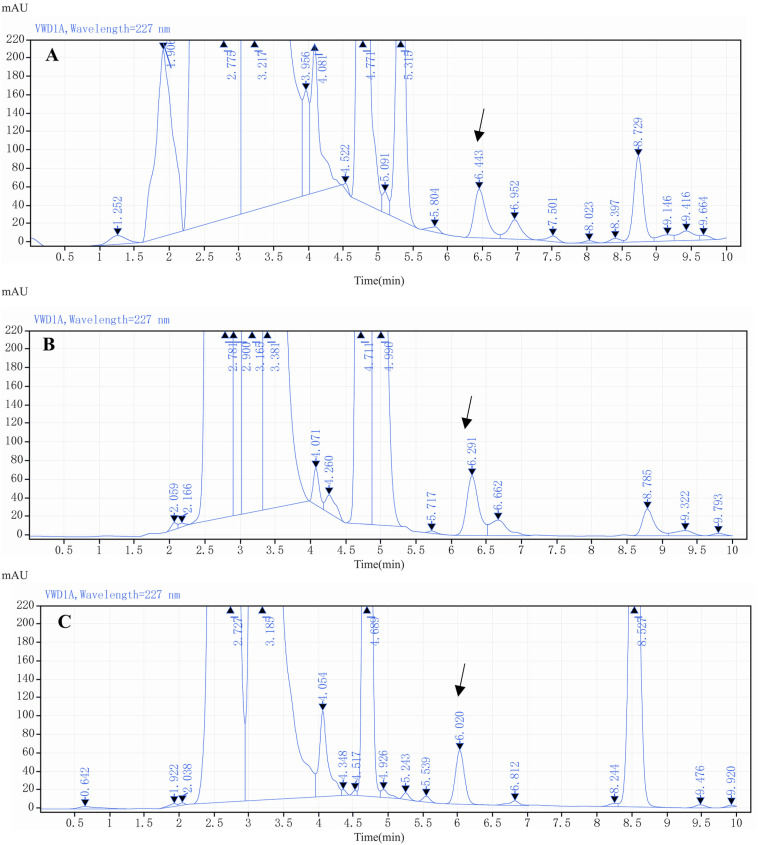
The chromatogram of LIN derivative separated by different chromatographic columns. (**A**) Agilent ZORBAX RX-C8 (250 mm × 4.6 mm i.d., 5 μm); (**B**) Agilent ZORBAX SB-C8 (150 mm × 4.6 mm i.d., 3.5 μm); (**C**) Agilent ZORBAX XBD- C18 (250 mm × 4.6 mm i.d., 5 μm).

LIN is a type of lincosamide antibiotic with high polarity, but it became less polar when it was derivatized by attaching four benzene rings to the hydroxyl group during derivatization. Thus, reversed-phase C18 and C8 columns were chosen for the analysis of derivatized products, as these columns are hydrophobic and able to retain nonpolar molecules commonly used in drug analysis [38,39]. This study compared the separation performance of three different columns—Agilent ZORBAX RX-C8 (250 mm × 4.6 mm i.d., 5 μm), Agilent ZORBAX SB-C8 (150 mm × 4.6 mm i.d., 3.5 μm) and Agilent ZORBAX XBD-C18 (250 mm × 4.6 mm i.d., 5 μm) (Agilent Technologies Inc., USA)—for LIN derivatives. The result showed that the target peak of Agilent ZORBAX XBD-C18 column had the highest resolution of 2.24, an EP number of theoretical shelves of 10,213.51 and a symmetry factor of 0.90; although the peak area was relatively low in Figure 9C because the target peak of the other columns showed a high peak area due to poor resolution. Therefore, Agilent ZORBAX XBD-C18 column (250 mm × 4.6 mm i.d., 5 μm) was selected as the column for analysis and detection, producing sharper peaks with better resolution from impurities and no peak tailing for the derivatives (Figure 9).

### 2.4. Method Validation

LIN was detected in blank pig liver and muscle; chicken kidney, liver and egg; cow fat, liver and milk; and goat muscle, liver and milk at concentrations within 3000 µg/kg. As shown in Table 1, the linear correlation between the peak area and concentration was strong (R^2^ = 0.9912–0.9994) across the range of LOQ-3000 µg/kg.

The recovery of LIN in spiked samples at three different concentrations (LOQ, MRL, and 2× MRL) ranged from 71.11% to 98.30%. The intra-day and inter-day RSD were 1.5–13.4% and 1.9–12.1%, respectively. The method was validated according to the criteria for chromatographic analysis and also demonstrated good accuracy and precision (Table 2).

**Figure 10:**
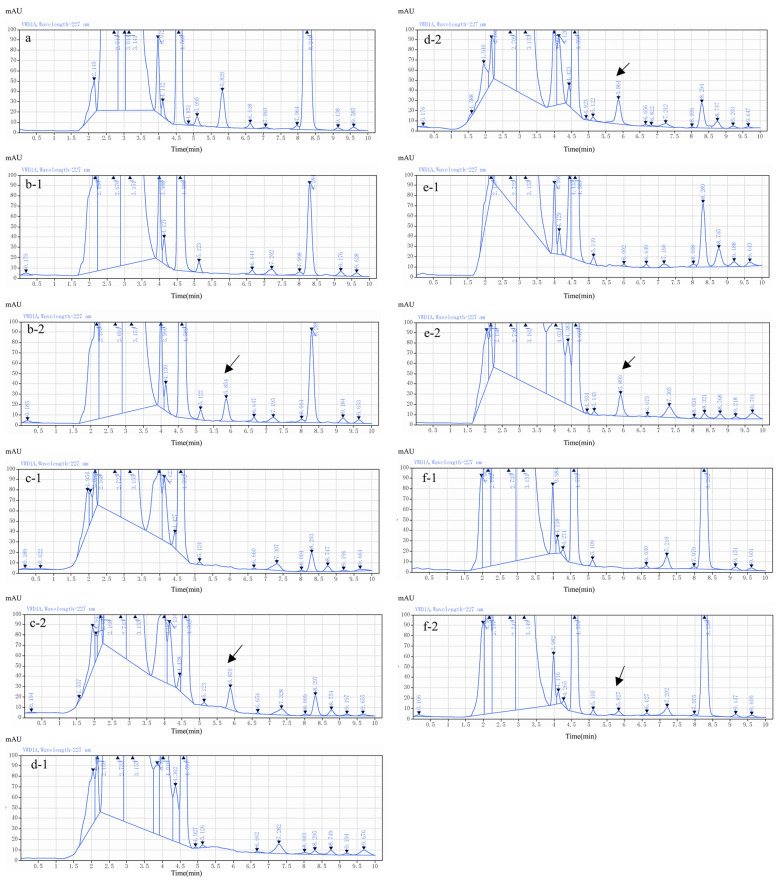

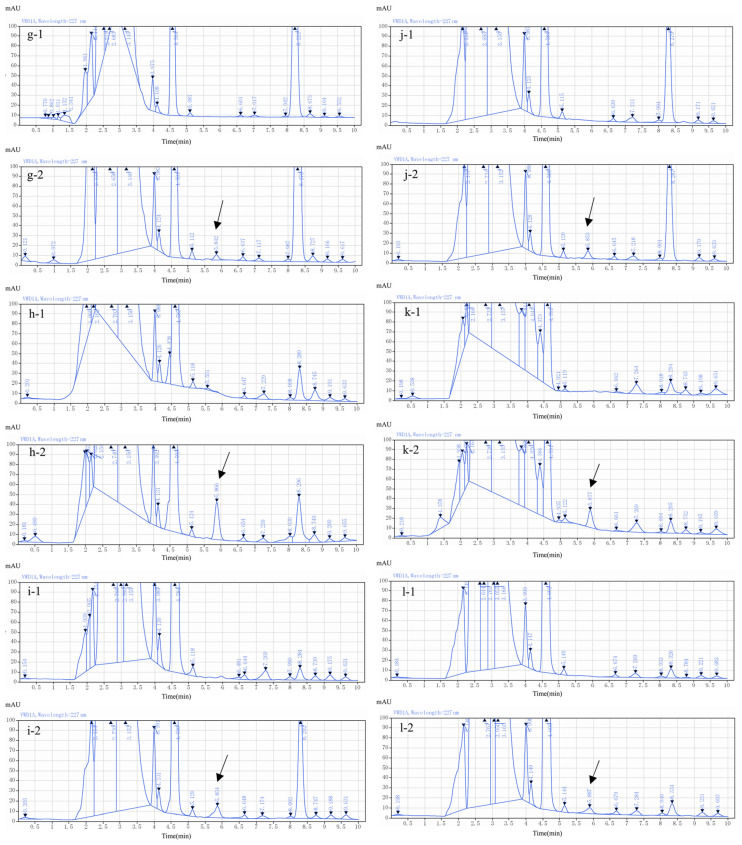
Representative chromatograms. (**a**) LIN standard solution; (**b-1**) blank pig muscle; (**b-2**) spiked pig muscle (200 μg/kg); (**c-1**) blank pig liver; (**c-2**) spiked pig liver (500 μg/kg); (**d-1**) blank chicken liver; (**d-2**) spiked chicken liver (500 μg/kg); (**e-1**) blank chicken kidney; (**e-2**) spiked chicken kidney (1000 μg/kg); (**f-1**) blank chicken egg; (**f-2**) spiked chicken egg (50 μg/kg); (**g-1**) blank cow fat; (**g-2**) spiked cow fat (50 μg/kg); (**h-1**) blank cow liver; (**h-2**) spiked cow liver (500 μg/kg); (**i-1**) blank cow milk; (**i-2**) spiked cow milk (150 μg/kg); (**j-1**) blank pig muscle; (**j-2**) spiked pig muscle (200 μg/kg) (**k-1**) blank goat liver; (**k-2**) spiked goat liver (500 μg/kg); (**l-1**) blank goat milk; (**l-2**) spiked goat milk (150 μg/kg).

The samples of different matrices (pig liver and muscle; chicken kidney, liver and egg; cow fat, liver and milk; and goat muscle, liver and milk) were processed following the session “Sample preparation and extraction” to obtain blank matrices and spiked samples for analysis. The chromatograms of the blank and spiked samples showed a sharp target peak without interfering peaks at approximately 6 min. These findings indicate the good sensitivity and selectivity of the method (Figure 10).

Finally, compared with the latest standard methods for lincomycin residue in animal-derived food in China—the National Standard of the People’s Republic of China GB 29685-2013 [40]—a gas chromatograph tandem mass spectrometry for lincomycin detection, this method was validated and applied to larger number of sample matrices. Besides, HPLC were more widely used and cost-effective equipment, fulfilling detection requirements; although the sensitivity is relatively low. Therefore, the development of HPLC for lincomycin residue detection was valuable.

## 3. Materials and Methods

The LIN standard (purity > 84.9%) was obtained from the China Veterinary Drug Supervision Institute (Beijing, China). HPLC-grade acetonitrile, methanol, phosphoric acid, and analytical-grade hexane and acetonitrile were obtained from Thermo Fisher Technology Co., Ltd (Dongguan, China). HPLC-grade potassium dihydrogen phosphate and anhydrous acetonitrile were obtained from Shanghai Macklin Biochemical Co., Ltd. Octadecylsilyl (C18) and primary-secondary amine (PSA) were obtained from Anpel Laboratory Technologies Inc (Shanghai, China). Ultrapure water was from Milli-Q Millipore Water System (Millipore Corp., Bedford, MA, USA) throughout the study. *p*-toluene sulfonyl isocyanic acid (PTSI) was obtained from Sigma (Berlin, Germany).

### 3.1. Solutions

LIN standard solutions were prepared in methanol at 1 mg/mL and were stably stored for one month at −20 °C. Working standard solutions of LIN were prepared as follows: 1 mg/mL total stock solution was accurately diluted with methanol to 100 μg/mL and 10 μg/mL for future use.

Saturated n-hexane was prepared by mixing n-hexane and acetonitrile, and allowing them to separate into two layers. PTSI (30%) was made up by mixing PTSI with anhydrous acetonitrile (30:70, *v*/*v*), and was used immediately.

### 3.2. Materials

Sample purification was performed using C18 and PSA powder, C18 SPE (Cartridge, 60 mg, 3 mL) and nylon syringe filters (0.22 μm, 13 mm) (Anpel Laboratory Technologies, Inc., Shanghai, China).

Tissue, milk and egg samples were collected from antibiotic-free animals at nearby farms. All the above samples were fully homogenized and stored at −20 °C until analysis.

### 3.3. Instrumentation and HPLC Conditions

Analysis was performed using an Agilent system (Agilent 1260, Agilent Technologies, USA). The chromatographic column used was a ZORBAX Eclipse XDB-C18 column (4.6 mm × 250 mm i.d., 5 μm). The mobile phase consisted of aqueous potassium dihydrogen phosphate (0.02 M containing 0.018% phosphoric acid, pH = 4.0) and acetonitrile (40:60, *v*/*v*) at 30 °C. At 1 mL/min flow rate, 10 μL of sample was injected and analytes were detected at 227 nm.

### 3.4. Sample Pretreatment

Sample preparation and extraction. A 5 g blank matrix sample was put into each 50 mL centrifuge tube and spiked with the LIN working standard solution at three different spiking concentrations, according to their MRL. For pig liver and muscle; chicken kidney and liver; cow liver and milk; and goat muscle, liver and milk, the three concentration levels were set at 60 ng/g, MRL and 2× MRL, and these three concentrations levels were 30 ng/g, MRL and 2× MRL for cow fat and eggs. The MRL of each type of sample in China is shown in Table 3.

In addition to cow fat, for the other tissues (pig liver and muscle; chicken kidney and liver; cow liver and milk; goat muscle, liver and milk; and eggs), 10 mL of acetonitrile was added to each tube. Then, the mixture was vortexed for 2 min and ultrasonicated for 10 min. Subsequently, the mixture was subjected to 7500× *g* of centrifugation for 10 min, and the transparent liquid on top of the sediment was moved to 15 mL centrifuge tube. This step of extraction was done once more, and the supernatants were merged into one centrifuge tube. The total extract was evaporated to a volume of approximately 2 mL under a stream of air at 45 °C.

For cow fat, 20 mL of n-hexane was added to each 50 mL centrifuge tube with 5 g spiked cow fat and shaken for 10 min to dissolve the fat. Subsequently, 20 mL of 50% acetonitrile (acetonitrile v/water v, 50:50) was added and shaken for 10 min. Then, the mixture was subjected to 7500× *g* of centrifugation for 10 min at 4 °C. Subsequently, the lower aqueous phase was sucked with pipe and transferred to a new centrifuge tube, and the solution was evaporated to a volume of approximately 10 mL under air at 45 °C.

Sample purification For most tissues (pig liver and muscle; chicken kidney and liver; cow liver and milk; goat muscle, liver and milk; and eggs), after extraction, 5 mL of pure water and 5 mL of saturated n-hexane were successively mixed with the concentrated extract. Subsequently, C18 and PSA powder were added to absorb impurities. The usage was as followed: 150 mg of C18 and 150 mg of PSA powder for pig muscle, cow fat and milk, and goat muscle, fat and milk; 300 mg of C18 and 300 mg of PSA powder for pig liver, chicken liver and kidney, cow liver, goat liver, and eggs. The mixture was vortexed for 1 min and centrifugated at 13,000× *g* for 5 min. The supernatant (saturated n-hexane) and the middle layer (insoluble impurities) were removed and discarded. The purification step was repeated two or three times depending on the amount of insoluble impurities.

For cow fat, 5 mL of saturated n-hexane was added and mixed with the extract to remove the remaining fat and impurities. The mixed solution was vortexed for 2 min and centrifuged at 13,000× *g* for 5 min at 4 °C. The supernatant (n-hexane) and the middle layer (insoluble impurities) were removed and discarded.

The final aqueous phase of all types of samples was retained and transferred to a new 10 mL centrifuge tube for future use. The SPE cartridge was conditioned with methanol (3 mL) and equilibrated with ultrapure water (3 mL). All of the above extracts were loaded onto an SPE column and allowed to completely pass through the column by gravity. Six milliliters of ultrapure water was loaded for washing. Finally, 3 mL of methanol was loaded to elute LIN. The eluate was collected in a 10 mL centrifuge tube and evaporated to dryness at 45 °C.

Derivatization. A quantity of 100 μL anhydrous acetonitrile and 100 μL 30% PTSI were added to the tube containing the residue of the eluate. The mixture was vortexed for 30 s and reacted in the dark at room temperature for 30 min. The reaction was then stopped by adding 100 μL of methanol. Subsequently, the tubes with derivatized products were vortexed for 30 s and evaporated to dryness under the stream of nitrogen at 45 °C. Finally, the derivatized products were concentrated, redissolved in the mobile phase (400 μL) and filtered through a filter for HPLC analysis [17].

### 3.5. Method Validation

The analytical performance of HPLC-UVD was measured by its selectivity, linearity, limit of detection (LOD), limit of quantification (LOQ), accuracy and precision [41]. The selectivity was examined by the significant difference of chromatograms of blank samples and spiked samples.

The analytical linearity of the standard curve was measured by the coefficient of determination (R^2^). Quantitative analysis was conducted with an external standard and performed using a standard curve generated from a series of spiked blank samples prepared in the same matrix [42]. The standard curve was constructed by serial dilutions (3, 5, 10, 25, 40 and 60 μg/mL) of the LIN working solution for pig liver and muscle; chicken kidney and liver; cow liver and milk; goat muscle, liver and milk; and by serial dilutions (1.5, 2.5, 4, 5, 7.5 and 10 μg/mL) for or cow fat and eggs. In total, 100 microliters of LIN working standard solution was added according to matrices for analysis of the different tissues, milk and eggs. The concentration and corresponding peak area of the standard curve were analyzed via regression, and the analysis was accurate at R^2^ > 0.99 [43].

The chromatographic peak areas (y) for the LIN derivatives showed a strong linear relationship with increasing LIN concentration (x). The sensitivity was indicated by Δy/Δx of the calibration curve. The LOD and LOQ values of the analysis were determined by analyzing blank extracts of different tissues, milk and eggs spiked with the LIN working solution. The LOD and LOQ of this HPLC method were calculated as the concentrations with signal/noise ratios of 3:1 and 10:1, respectively [44,45]. Six replicates of the spiked samples at each concentration level were analyzed.

The analytical accuracy of the proposed method was evaluated based on the recovery, and the precision was evaluated using the relative standard deviation (RSD). To obtain recovery and RSD, the data of the samples spiked at the concentrations of the LOQ, MRL and 2 × MRL were analyzed as follows. The concentrations detected in the spiked samples were calculated according to Formula (1). The recovery rate of each sample was calculated as the ratio of the detected concentration to the spiked concentration. The number of replicates for each concentration level of each type of matrix was six. The ratio of the standard deviation (SD) to the average recovery of these six replicates was intra-day RSD and the ratio of the SD to the matrix the spiked at 3 different levels on 3 different days was calculated as inter-day RSD [46].

(1) $$X=\frac{A-b}{a}*\frac{V}{m}$$

X—residue of LIN in spiked sample, μg/kg or μg/L;

A—chromatographic peak area of LIN in the sample solution after sample treatment;

b—the intercept of regression equation which was obtained from standard curve;

a—the slope of the regression equation which was obtained from standard curve.

m—mass or volume of the sample, kg or L;

V—the total volume of derivatized product after redissolving, L.

## 4. Conclusions

In this study, we developed a reliable and cost-effective HPLC-UVD method to detect LIN in animal-derived food (pig liver and muscle; chicken kidney, liver and egg; cow fat, liver and milk; and goat muscle, liver and milk). Based on our previous studies, this method was well refined and applied to more types of matrices in this work. The HPLC method was used to analyze limited animal matrices, and this study provided further data on the detection of LIN residues in other foods of animal origin. The optimization—especially for extraction and HPLC conditions—was able to analyze livers, kidneys and eggs, the most challenging matrices; and significantly decrease the LOD and LOQ of LIN in various matrices by HPLC, with high sensitivity, good linearity, selectivity, precision and accuracy. The method achieved excellent analytical performance, good separation efficiency and great purification efficiency compared with other methods, fulfilling the criteria for drug residue and actual detection requirements. We succeeded in extracting lincomycin from fat and eliminating the interference of impurities and pigment from livers, kidneys and eggs. HPLC was developed and validated, being applied to the identification and determination of lincomycin in unprecedented numbers and types of matrices. In addition, derivatization reactions between LIN and PTSI make the detection of drugs with weak UV absorption possible and provide a new idea for detecting LIN or other drugs with weak UV absorption in food, water or environment.

## Figures and Tables

**Table 1:** Linear level of LIN in 11 animal matrices.

Animal	Type	Batch	Linear Range (μg/kg)	Calibration Equations	R^2^
Pig	Liver	1	60~1200	y = 0.731x + 10.36	0.9948
		2	60~1200	y = 0.899x + 0.400	0.9993
		3	60~1200	y = 1.050x + 9.441	0.9978
	Muscle	1	60~1200	y = 1.176x + 8.105	0.9995
		2	60~1200	y = 1.009x + 24.099	0.9986
		3	60~1200	y = 1.092x + 20.015	0.9989
Chicken	Kidney	1	60~3000	y = 0.970x + 3.134	0.9971
		2	60~3000	y = 0.915x + 26.74	0.9929
		3	60~3000	y = 0.720x + 13.94	0.9938
	Liver	1	60~1200	y = 1.086x − 11.53	0.9962
		2	60~1200	y = 0.994x + 17.58	0.9945
		3	60~1200	y = 1.076x + 20.76	0.9960
	Eggs	1	30~200	y = 0.969x + 3.134	0.9971
		2	30~200	y = 0.915x + 26.743	0.9929
		3	30~200	y = 0.720x + 13.937	0.9938
Cow	Fat	1	30~200	y = 1.079x − 0.670	0.9993
		2	30~200	y = 1.205x − 1.801	0.9958
		3	30~200	y = 1.341x + 10.84	0.9906
	Liver	1	60~1200	y = 1.160x + 17.07	0.9946
		2	60~1200	y = 0.837x − 0.631	0.9983
		3	60~1200	y = 0.728x + 9.432	0.9948
	Milk	1	60~1200	y = 0.911x − 1.3203	0.9980
		2	60~1200	y = 0.971x + 15.73	0.9969
		3	60~1200	y = 1.007x + 8.696	0.9917
Goat	Muscle	1	60~1200	y = 1.107x + 0.5321	0.9982
		2	60~1200	y = 1.033x − 4.560	0.9986
		3	60~1200	y = 1.071x + 26.805	0.9986
	Liver	1	60~1200	y = 0.788x − 5.920	0.9989
		2	60~1200	y = 1.038x + 26.80	0.9994
		3	60~1200	y = 0.874x − 11.96	0.9917
	Milk	1	60~1200	y = 1.018x + 6.593	0.9979
		2	60~1200	y = 1.125x + 4.823	0.9995
		3	60~1200	y = 1.045x + 4.727	0.9996

**Table 2:** Recovery and precision of LIN in 11 animal matrices.

Matrix	Spiked Concentration (μg/kg)	Recovery (X ± SD, %, n = 6)			Intra-Day RSD (%, n = 6)			Inter-Day RSD (%, n = 18)
		1	2	3	1	2	3	
Pig muscle	60	86.9 ± 6.3	95.0 ± 4.1	81.8 ± 5.3	7.3	4.3	6.4	8.5
	200	97.5 ± 2.1	84.9 ± 2.0	81.0 ± 10.2	2.1	2.4	12.6	10.5
	400	96.6 ± 2.3	97.1 ± 1.4	97.2 ± 1.9	2.3	1.5	2.0	1.9
Pig liver	60	90.0 ± 6.3	89.1 ± 9.2	94.1 ± 5.0	7.0	10.4	5.0	7.6
	500	88.7 ± 4.4	81.2 ± 6.0	89.6 ± 5.4	4.9	7.4	6.1	7.3
	1000	85.1 ± 4.4	75.1 ± 2.7	74.1 ± 2.9	5.2	3.6	3.9	7.7
Chicken liver	60	88.1 ± 4.9	91.2 ± 5.8	88.3 ± 5.5	5.6	6.4	6.2	5.9
	500	77.4 ± 3.7	73.7 ± 1.9	81.3 ± 5.8	4.8	2.6	7.1	6.5
	1000	86.0 ± 3.2	91.6 ± 4.5	83.7 ± 3.6	3.8	4.9	4.3	5.7
Chicken kidney	60	88.0 ± 6.7	76.9 ± 3.2	89.6 ± 7.9	7.6	4.2	8.8	9.7
	500	80.5 ± 10.1	74.8 ± 3.9	89.9 ± 5.9	12.2	5.2	6.5	11.1
	1000	75.4 ± 3.3	73.5 ± 1.9	85.8 ± 5.2	4.3	2.6	6.0	8.4
Chicken egg	30	82.0 ± 5.1	80.1 ± 8.2	76.4 ± 6.7	6.2	10.3	8.8	9.3
	50	81.5 ± 8.5	80.6 ± 7.7	89.1 ± 7.9	10.4	9.5	8.8	10.8
	100	79.2 ± 3.5	80.8 ± 11.3	90.6 ± 7.2	4.4	13.9	7.9	12.1
Cow fat	30	88.0 ± 9.8	84.7 ± 8.4	84.0 ± 5.3	11.1	10.0	6.3	9.1
	50	92.0 ± 4.2	91.2 ± 6.4	79.2 ± 8.0	4.5	7.0	11.2	10.0
	100	95.1 ± 1.9	89.3 ± 3.3	81.5 ± 5.0	2.0	3.7	6.1	7.5
Cow liver	60	87.3 ± 8.5	89.2 ± 5.8	88.6 ± 6.3	9.7	6.5	7.1	7.5
	500	87.5 ± 8.5	82.4 ± 5.8	92.2 ± 3.9	9.7	7.0	4.3	8.3
	1000	73.9 ± 6.2	78.6 ± 7.7	86.8 ± 5.3	8.5	9.8	6.1	10.3
Cow milk	60	89.0 ± 9.0	91.0 ± 3.0	84.7 ± 5.6	10.1	3.3	6.6	7.4
	150	93.5 ± 5.3	80.5 ± 2.3	87.3 ± 7.1	5.6	2.9	8.1	8.4
	300	77.6 ± 8.6	76.9 ± 5.3	75.1 ± 7.8	11.0	7.7	10.5	10.7
Goat muscle	60	89.6 ± 4.9	89.0 ± 2.4	88.9 ± 7.1	8.0	2.7	9.1	5.4
	100	90.3 ± 7.2	95.3 ± 2.5	83.6 ± 10.2	8.0	2.6	12.2	9.4
	200	87.8 ± 9.1	90.4 ± 5.0	79.3 ± 5.5	10.4	5.6	6.9	9.4
Goat liver	60	75.6 ± 5.1	79.1 ± 4.9	93.9 ± 6.6	6.8	6.2	7.0	11.7
	500	80.6 ± 8.0	84.2 ± 5.1	81.6 ± 3.2	9.9	6.1	3.9	6.9
	1000	82.2 ± 6.3	79.8 ± 6.1	75.6 ± 4.3	7.6	7.6	5.7	7.5
Goat milk	60	82.4 ± 11.0	93.4 ± 4.9	84.7 ± 5.6	13.4	5.2	5.1	10.2
	150	89.5 ± 6.5	95.8 ± 3.0	87.3 ± 7.1	7.3	3.1	11.4	8.6
	300	88.1 ± 5.5	94.3 ± 6.1	75.1 ± 7.8	6.2	6.4	5.0	6.4

**Table 3:** National food safety standard maximum residue limits for veterinary drugs in foods and limits of detection and quantification of LIN in food of animal origin.

Animal	Type	MRL (μg/kg)	LOD (μg/kg)	LOQ (μg/kg)
Pig	Liver	500	40	60
	Muscle	200	30	60
Chicken	Liver	500	40	60
	Kidney	500	40	60
	Eggs	50	25	40
Cow	Fat	50	25	40
	Liver	500	40	60
	Milk	150	30	60
Goat	Muscle	200	30	60
	Liver	500	40	60
	Milk	200	30	60

## Data Availability

The original contributions presented in the study are included in the article, further inquiries can be directed to the corresponding authors.
